# Clinical Presentation and Outcomes of Acute Disseminated Encephalomyelitis in Adults Worldwide: Systematic Review and Meta-Analysis

**DOI:** 10.3389/fimmu.2022.870867

**Published:** 2022-06-09

**Authors:** Kunyi Li, Maolin Li, Lan Wen, Qiancheng Wang, Xin Ding, Jian Wang

**Affiliations:** ^1^ Department of Neurology, the Second People’s Hospital of Chengdu, Chengdu, China; ^2^ Department of Neurology, People’s Hospital of Deyang City, Deyang, China; ^3^ Department of Neurosurgery, West China Hospital, Sichuan University, Chengdu, China; ^4^ Department of Neurology, Chongqing North-Kuanren General Hospital, ChongQing, China

**Keywords:** ADEM, clinical feature, outcome, meta, adult

## Abstract

**Background:**

Acute disseminated encephalomyelitis (ADEM) is a rare demyelinating disorder that is often misdiagnosed. To improve early diagnosis, we performed a systematic review and meta-analysis of clinical features, outcomes for ADEM in adults.

**Methods:**

The PubMed, Embase, Web of Science and Cochrane Library databases were searched for studies reporting the clinical features of adults with ADEM between January 1990 and May 2021. A random-effects meta-analysis model was used to pool data on clinical features and functional outcomes.

**Results:**

Twelve studies examining 437 adults with ADEM met the inclusion criteria. Overall, the clinical features and diagnostic findings observed in more than two-thirds of the patients were white matter lesions [87.1%, 95% confidence interval (CI)=75-95.6], polyfocal onset (80.5%, 95% CI=50.5-98.9) and pyramidal signs (68.7%, 95% CI =40.0-91.9). The mortality rate was 7.8% (95% CI = 3.3–13.5), and the risk of residual deficits was 47.5% (95% CI = 31.8–63.4).

**Conclusions:**

Adults with ADEM had worse outcomes than children. Clinicians should maintain high clinical suspicion for patients presenting with certain clinical features and diagnostic findings.

## Introduction

Acute disseminated encephalomyelitis (ADEM) is a rare immune-mediated inflammatory demyelinating disorder of the central nervous system (CNS) (1, 2) that was first described in a patient after smallpox infection 250 years ago (3). Although it occurs at all ages, because it is commonly preceded by viral infections or vaccinations, ADEM is more common in children than in adults (2). With the ongoing COVID-19 pandemic and subsequent vaccination use, the incidence of ADEM may increase (4).

Early and accurate diagnosis is important to start prompt treatment and improve outcomes. Due to the lack of specific biomarkers, clinical features play a vital role in the diagnosis of ADEM (5). Other demyelinating disorders, such as multiple sclerosis (MS), may be indistinguishable from ADEM at initial presentation (6). Recently, the International Pediatric Multiple Sclerosis Study Group (IPMSSG) proposed a consensus definition of ADEM for children (7). Due to discrepancies in clinical features between children and adults, this consensus definition may not be suitable for diagnosis in adults. To date, no specific diagnostic criteria have been established for adults.

Recently, several studies have focused on the clinical features of ADEM in adults and found that adult patients had worse outcomes than children (8, 9). However, most of these studies were single-centre studies with small sample sizes. Worldwide data on the clinical features of ADEM in adults are still inconclusive. A systematic evaluation using an evidence-based approach is urgent. To better characterize this rare clinical entity, we conducted a systematic meta-analysis to investigate the clinical features and outcomes of ADEM in adults.

## Methods

### Literature Search

Two review authors (L.W, Q.C. W) independently searched the PubMed, Embase, Web of Science and Cochrane Library databases. The study search was limited to articles published in English between January 1990 and May 2021. The search terms used in each database included acute disseminated encephalomyelitis and adults (**Supplementary Material I**).

### Eligibility Criteria and Data Extraction

All eligible studies were cohort studies with ADEM patients older than 14 years reporting clinical features and outcomes. Each study reported at least 5 patients. We excluded patients with central nervous system infection, vasculitis, or other autoimmune diseases. Data extracted from eligible studies included the first author, publication year, country, study design, population demographics, clinical features, imaging findings, CSF results, treatment and outcomes including the death and residual deficits. Two study investigators (K.Y.L and M.L.L) independently extracted data from selected articles. Disagreement or uncertainties were resolved by consensus with a third investigator (L.W). To deal with missing data, the study authors were contacted, when necessary.

### Quality Assessment

Quality rating of the included studies was performed through the National Institutes of Health Quality Assessment Tool for Case Series Studies (10, 11). Articles were rated as good, fair, or poor independently by two investigators (K.Y.L and M.L.L). If ratings were different, two investigators discussed the articles to reach an agreement.

### Certainty of Evidence

Certainty of evidence was evaluated by the Grading of Recommendations Assessment, Development, and Evaluation (GRADE) approach. The quality of evidence was rated as high, moderate, low, or very low (12).

### Statistical Analysis

The crude frequencies of clinical presentations, and outcomes were first computed for each study and then double-arcsine transformed using the Freeman-Tukey method (13). Meta-analyses with the command metaprop and metan were used to calculate pooled estimates of proportions (95% CI) and pooled estimates of means (95%CI) of clinical presentations, and outcomes. Heterogeneity was tested using Cochran’s Q statistic, and a *p* value below 0.1 indicated significant heterogeneity. The extent of heterogeneity was quantified using the I^2^ statistic (14). Because of substantial heterogeneity among the included studies, a random-effects model was used to adjust for this prior to pooling the study-specific frequencies of clinical presentations, diagnostic findings, and functional outcomes. To determine whether a single study had a disproportional influence on the pooled results, a “leave-one-out” sensitivity analysis was performed to calculate the robustness of the pooled results (15). To investigate the potential sources of heterogeneity, we performed subgroup analyses according to location (Europe/Asia/South America) and follow-up time (≤6 months/>6 months). Publication bias was assessed by visual inspection of funnel plots, and tested for significance by using Begg’s rank correlation test and Egger’s regression test (16, 17). All *p* values were two-sided, and a *p* value below 0.05 was considered statistically significant. All statistical analyses were performed using STATA version 14.0 (STATA, College Station, TX).

## Results

### Study Selection and Characteristics

**Figure 1:**
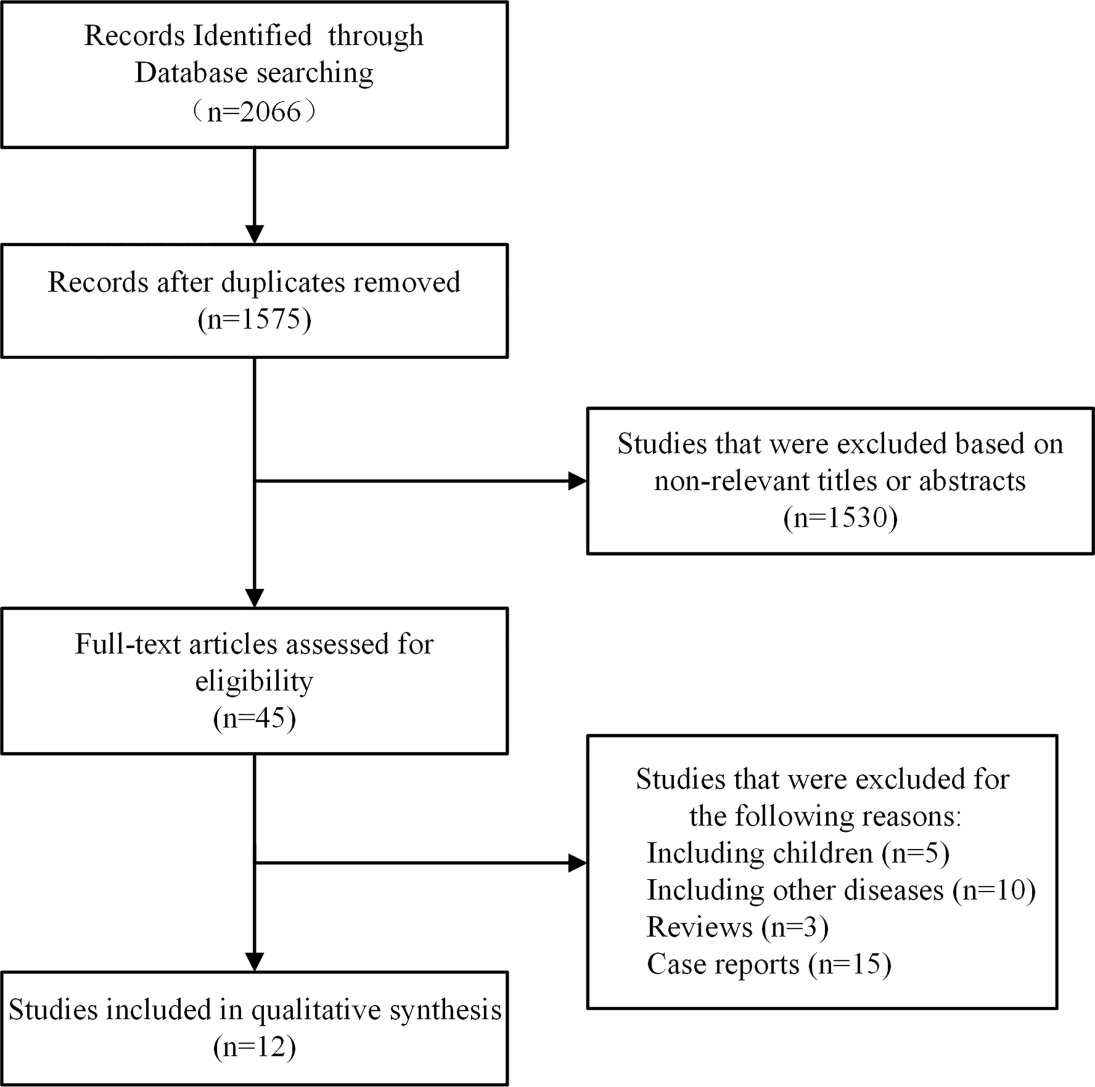
Flowchart of the included studies.

This systematic review and meta-analysis was conducted following the PRISMA guidelines (**Supplementary Material II**). **Figure 1** shows the study selection process and the results of the meta-analysis. The initial search strategy identified 2066 articles. After removing 491 duplicates, a total of 1575 articles were reviewed for relevance by titles and abstracts, and 45 articles remained for further investigation. After detailed assessments, 12 articles (8, 9, 18–27) met the inclusion criteria and were included in this meta-analysis. Forest plots of all the meta-analyses were available in **Supplementary Material III**.

### Quality Assessment

The included studies were rated as good [8(66.6%)] or fair [4 (33.3%)] quality, which were assessed by the National Institutes of Health Quality Assessment Tool for Case Series Studies.

### Certainty of Evidence

The qualities of the evidence were assessed by GRADE guidelines. Observational studies started with a “low quality” rating, and the quality of evidence was downgraded for the “imprecision”. Thus, the overall qualities of the evidence were rated as “very low” (**Supplementary Material IV**).

**Table 1** summarizes the baseline characteristics of all included studies. In total, 12 included studies were published between 2000 and 2020 and involved a total of 437 cases, with 11 retrospective studies and 1 prospective study. Nine were single-centre studies, and 3 were multicentre studies. Of these studies, 4 were from Europe, 4 were from Asia, 1 was from Oceania, 1 was from North America, 1 was from South America and 1 was from both Asia and North America. A total of 41.7% of patients were male, and the mean age was 37.1 ± 23.1 years. A total of 45.7% of patients met the IPMSSG criteria for ADEM at presentation (7).

**Table 1:** Characteristics of included studies in the meta-analysis.

				Total	Mean age	Treatment		
First author, year	Period of study	Country	Study design	cases	(years)	Steroids	Plasma exchange	Intravenous immunoglobulin
S. Schwarz 2001 (21)	1988-1999	German	Retrospective	26	38.2	26/26	Not reported	Not reported
C-H Lin 2007 (8)	1991-2005	China	Retrospective	30	50	30/30	1/30	5/30
Diederik L.H. Koelman 2016 (18)	1985-2014	America	Retrospective	106	37.4	Not reported	13/106	Not reported
Uma Sundar 2012 (22)	Not report	India	Prospective	29	33	25/29	Not reported	Not reported
D. L. H. Koelman 2017 (20)	1992–2015	China et al	Retrospective	67	39	Not reported	Not reported	17/67
Jérôme de Seze 2007 (23)	1995-2005	French	Retrospective	35	35.9	35/35	1/35	3/35
Hong-Qi Yang 2016 (25)	2003-2013	China	Retrospective	42	33.5	35/42	Not reported	7/42
JN Panicker 2010 (26)	1999-2004	India	Retrospective	38	30.1	Not reported	Not reported	Not reported
D. Imbesi 2012 (27)	2002-2004	Italy	Retrospective	6	43.4	Not reported	Not reported	Not reported
IA Ketelslegers 2011 (9)	1998-2008	Netherlands	Retrospective	25	40.8	21/25	Not reported	Not reported
Peter Höllinger 2002 (19)	1998-2001	Switzerland	Retrospective	10	37.6	8/10	2/10	3/10
Marcell Pourbaix 2020 (24)	1999-2016	Brazil	Retrospective	23	30.8	23/23	Not reported	2/23

### Characteristics at Initial Presentation

**Table 2** summarizes the clinical features provided by the 12 studies.

**Table 2:** Clinical features in patients with ADEM.

		Proportion		Heterogeneity	
	Number of patients	(95% CI)	*p*	I^2^,%	*p*
**Demographics and preceding events**					
Age (mean±SD)	437	37.1±23.1	<0.001	55.3	0.01
Sex, male	399	41.7 (35.3-48.3)	<0.001	32.6	0.138
Preceding infection	266	49.8 (33.6-66)	<0.001	81.7	<0.001
Upper respiratory tract infection	177	25.7 (12.2-41.7)	<0.001	77.2	<0.001
Acute gastroenteritis	146	8.7 (2.6-17.2)	<0.001	49.8	0.093
Preceding vaccination	220	2.9 (0-8.3)	0.032	54.8	0.024
Delay after infection or vaccinations episode (mean±SD,days)	139	12.5±21.7	<0.001	79.9	0.001
**Symptoms and signs**					
Polyfocal onset	295	80.5 (50.5-98.9)	<0.001	96.3	<0.001
Pyramidal signs	64	68.7 (40.0-91.9)	<0.001	77.4	0.004
Motor deficits	383	63.4 (56.9-69.6)	<0.001	33.7	0.148
Gait abnormality	183	52.0 (37.0-66.8)	<0.001	66.6	0.05
Brainstem symptom	109	46.7 (25.1-69.0)	<0.001	81.8	<0.001
Encephalopathy	237	43.7 (33.6-54.1)	<0.001	50.5	0.088
Sphincter dysfunction	155	40.1 (23.6-57.8)	<0.001	79.1	<0.001
Cranial nerve palsies	248	38.3 (31.3-45.5)	<0.001	15.6	0.315
Headache	311	38.2 (29.0-47.7)	<0.001	59.3	0.022
Sensory deficits	367	35.2 (23.8-47.5)	<0.001	79.8	<0.001
Fever	335	34.6 (20.9-49.5)	<0.001	84.2	<0.001
Ataxia	405	27.5 (23.1-32.1)	<0.001	0	0.664
Nausea/vomiting	248	27.3 (21.7-33.2)	<0.001	0	0.502
Spinal symptoms	91	26.6 (8.4-49.7)	<0.001	80	0.007
Seizures	437	13.6 (10.3-17.2)	<0.001	0	0.521
Meningeal signs	276	12.4 (7.6-18.1)	<0.001	28.2	0.213
Optic neuritis	405	11.7 (7.0-17.3)	<0.001	52.3	0.026
Extrapyramidal syndrome	107	11.3 (5.5-18.5)	<0.001	0	0.495
Aphasia	129	8.6 (3.4-15.5)	<0.001	24.5	0.265

SD, standard deviation.

Clear antecedent events (infectious event or vaccination) preceded the illness in half of the patients [51.7%, 95% confidence interval (CI) 38.2–65.0]. The interval between the preceding event and illness onset varied, ranging from 0 to 60 days (mean 12.5 ± 21.7 days). Preceding infections mostly involved the upper respiratory tract (25.7%, 95% CI 12.2-41.7) and less often involved the gastrointestinal tract (8.7%, 95% CI 2.6-17.2). Immunizations accounted for only 2.9% (95% CI 0.0-8.3) of cases.

**Figure 2:**
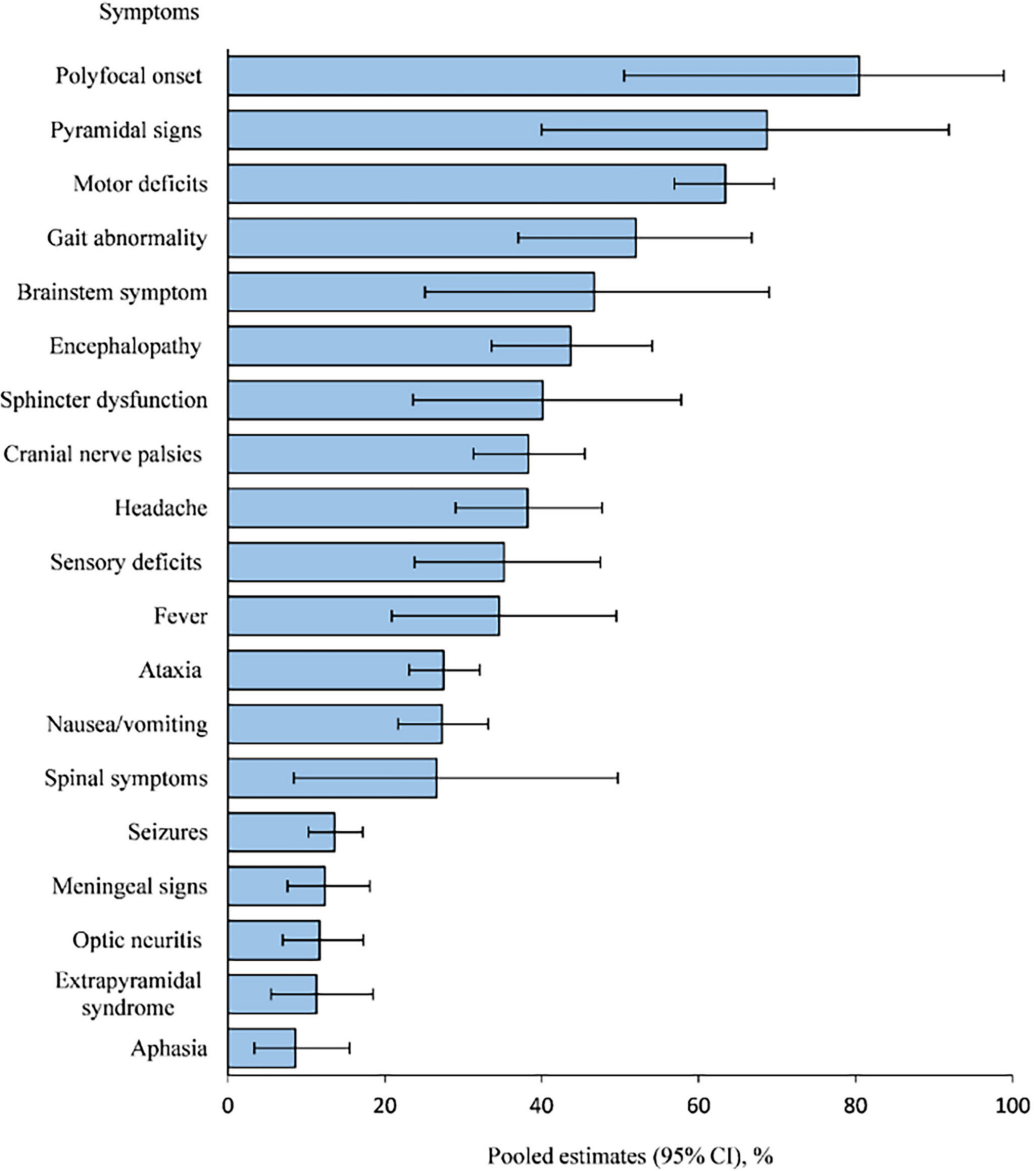
Symptoms and signs of patients with ADEM.

**Figure 2** shows the frequencies of symptoms and signs at presentation. Most patients (80.5%, 95% CI 50.5–98.9) had a polyfocal clinical presentation. The most common clinical features included pyramidal signs (68.7%, 95% CI 40.0-91.9), motor deficits (63.4%, 95% CI 56.9–69.6), gait abnormalities (52.0%, 95% CI 37.0-66.8), brainstem symptoms (46.7%, 95% CI 25.1-69.0) and encephalopathy (43.7%, 95% CI 33.6-54.1).

### MRI Features

A summary of the imaging findings of patients with ADEM is provided in **Table 3**. Among the patients who underwent MRI examinations, more than 90% of patients (91.6%, CI 75.0-95.6) presented with abnormal brain MRI findings. Gadolinium-enhancing lesions occurred in nearly 3/5 patients (58.0%, CI 33.6-80.7). The most frequent location of involvement was white matter (87.1%, CI 81.5-98.4), including periventricular white matter (43.2%, 95% CI 26.8-60.3) and subcortical areas (41.9%, 95% CI 31.8-52.2). In addition, an abnormal spinal cord was evident in nearly 41.6% (CI 14.4-51.2) of patients, including spinal cord lesions in > 2 segments in 1/3 of ADEM patients (31.6% CI 14.4-51.2).

**Table 3:** Diagnostic findings of patients at admission.

	Number of studies		Proportion		Heterogeneity	
	reporting	Number of patients	(95% CI)	*p*	I^2^,%	*p*
**Brain MRI findings**						
Abnormal brain	8	297	91.6 (81.5-98.4)	<0.001	79.3	<0.001
Abnormal white matter	2	62	87.1 (75-95.6)	<0.001	33.9	0.219
Abnormal periventricular white matter	10	388	43.2 (26.8-60.3)	<0.001	90.3	<0.001
Abnormal subcortical areas	5	129	41.9 (31.8-52.2)	<0.001	27	0.242
Abnormal corpus callosum	7	330	18.0 (8.6-29.6)	<0.001	82.2	<0.001
Abnormal brainstem	7	265	39.3 (23.7-56)	<0.001	83.9	<0.001
Abnormal deep gray matter	4	175	32.4 (21.6-44.1)	<0.001	49.1	0.117
Abnormal basal ganglia	5	153	30.7 (16.7-46.7)	<0.001	74.9	0.003
Abnormal cerebellum	9	317	29.8 (19.7-40.9)	<0.001	72.5	<0.001
Abnormal cortex	5	140	23.8 (14.1-34.9)	<0.001	50.5	0.089
Abnormal thalamus	3	91	9.9 (3.0-19.4)	<0.001	33.8	0.221
Mass effect	2	77	12.4 (4.5-23.5)	<0.001	43.3	0.184
Gadolinium-enhancing lesions	5	116	58.0 (33.6-80.7)	<0.001	84.6	<0.001
**Spinal MRI findings**						
Abnormal spinal cord	9	313	41.6 (26.0-58.1)	<0.001	85.3	<0.001
Spinal cord lesions >2 segments	2	28	31.6 (14.4-51.2)	<0.001	78.3	<0.001
**CSF analysis**						
Abnormal CSF	6	194	70 (54.8-83.3)	<0.001	75.8	<0.001
Pleocytosis(>5/μl)	5	214	51.8 (33.0-70.3)	<0.001	82.9	<0.001
Elevated protein (>45 mg/dL)	5	229	39.1 (14.1-67.4)	<0.001	93.5	<0.001
Lymphocytes predominant CSF	3	82	33.1 (17.6-50.5)	<0.001	53.7	0.115
Positive CSF OCBs	11	309	23.9 (12.1-37.8)	<0.001	81.3	<0.001

MRI, magnetic resonance imaging; CSF, cerebrospinal fluid; OCBs, oligoclonal bands.

### Laboratory Findings

CSF results were abnormal in 70.0% (95% CI 54.8–83.8) of adult patients with ADEM. Pleocytosis occurred in 51.8% (95% CI 33.0–70.3) of patients. CSF protein was increased (> 45 mg/dL) in 39.1% (95% CI 14.1-67.4) of patients. 23.9% (CI 12.1-37.8) of patients showed positive OCB results in CSF. Among the three included studies, 60 patients were tested for aquaporin-4 (AQP4) antibody in serum and all were negative (9, 18, 20).

### Treatment and Functional Outcomes

Treatments and patient outcomes are shown in **Table 1** and **4**. The mean duration of hospitalization was 23.1 days, ranging from 1 to 167 days, and 39.7% (95% CI 23.5-57.1) of patients required admission to the ICU. A total of 95.2% (95% CI 87.4-99.7) of patients were treated with corticosteroids, 16.4% (95% CI 9.2-24.9) were treated with intravenous immunoglobulin G (IVIg), and 7.3% (95% CI 2.0-14.7) were treated with plasma exchange (PLEX).

**Table 4:** Treatment and outcomes of patients with ADEM.

	Number of studies		Proportion		Heterogeneity	
	reporting	Number of patients	(95% CI)	*p*	I^2^,%	*p*
**Treatment**						
Corticosteroids treatment	8	220	95.2 (87.4-99.7)	<0.001	72.5	<0.001
Plasma exchange	4	181	7.3 (2.0-14.7)	<0.001	43	0.153
Intravenous immunoglobulin	5	177	16.4 (9.2-24.9)	<0.001	40.5	0.151
ICU admission	2	35	39.7 (23.5-57.1)	<0.001	0	0.448
Hospital stay (mean±SD,days)	5	163	23.1±23.2	<0.001	95.8	<0.001
**Outcomes**						
Death	9	247	7.8 (3.3-13.5)	<0.001	40.8	0.096
Residual deficits	8	218	47.5 (31.8-63.4)	<0.001	79.4	<0.001

ICU, intensive care unit; SD, standard deviation.

A total of 7.8% (95% CI 3.3-13.5) of patients died and nearly half of the patients (47.5%, 95% CI 36.8-63.4) suffered from residual deficits during the follow-up (2.8 ± 3.6 years). In addition, the recurrence of ADEM occurred in 7.2% (95% CI 2.0-20.8) of patients.

### Sensitivity Analysis of Mortality

The “leave-one-out” sensitivity analysis (**Supplementary Material V**) indicated that the mortality ranged from 6.3% (95% CI 2.8-10.7) to 9.8% (95% CI 5.5-11.5). Thus, no single study had a disproportional effect on the pooled results. Publication bias was not significant, based on visual inspection of funnel plots and the results of Begg’s test and Egger’s test (**Supplementary Material VI**).

**Table 5** summarizes the results of the subgroup meta-analysis of mortality. We determined region-specifc mortality in ADEM adults in Europe, Asia, and Americas. Patients living in Asia (14.5%) had higher mortality than those in other areas (0.4-5.0%). The follow-up time-specifc mortality in ADEM adults was 4.3% for the first 3 months, and 11.0% for more than 3 months.

**Table 5:** Subgroup analyses for mortality in ADEM adults.

				Heterogeneity
Subgroups	Categories	No. of studies	ES (%95CI)	I^2^ (%)
Location	Europe	4	5.5 (0.5-13.7)	0.0
	Asia	3	14.5 (5.9-25.6)	46.4
	Americas	1	0.0 (0.0-14.8)	-
Follow-up time	<=3months	3	4.3 (2.0-11.7)	34.7
	>3months	6	11.0 (5.1-18.5)	25.9

## Discussion

The lack of available discrete biomarkers remains a challenge in the diagnosis of ADEM. Currently, the diagnostic criteria of the International Paediatric Multiple Sclerosis Society Group (IPMSSG), which are evaluated by clinical and neuroimaging features, have been partly successful in diagnosing paediatric ADEM. Without alternative criteria for the adult population, the IPMSSG criteria have also been applied in adults in recent studies (28). However, the clinical and imaging profile of ADEM differs substantially according to age (29). Applying the IPMSSG criteria to all adults diagnosed with ADEM leaves more than half of cases without a diagnosis (18, 20). To guide clinical diagnosis and interventions for ADEM adults, we tried to combine our main findings of clinical features and the IPMSSG criteria to propose a summary of the key observations in ADEM adults (**Table 6**).

**Table 6:** A summary of the key observations in ADEM adults.

1. A first polyfocal, clinical central nervous system event with presumed inflammatory
demyelinating cause.
2. Clinical features:
1) Pyramidal signs 2) Brainstem symptoms
3) Encephalopathy that cannot be explained by fever
3. Antecedent events (infectious event or vaccination) can precede the illness.
4. Lesion characteristics on MRI:
1) MRI is abnormal during the acute (3 months) phase with diffuse, poorly demarcated,
large (>1– 2cm) lesions predominantly involving the white matter.
2) Deep grey matter, brainstem, cerebellum and spinal cord lesions can be present.
3) T1 hypointense lesions in the white matter are rare.
5. Exclusion of alternative diagnosis including other inflammatory demyelinating diseases and encephalitis.

Among 437 ADEM adults included in 12 studies, ADEM could occur in any age group, with a mean age at onset of 37.1 years. The ratio of males to females with ADEM changes with advancing age. A male predominance has been shown in most studies of paediatric cohorts (30, 31). For adults, we found that the ratio of females was higher than that of males, which is consistent with other inflammatory demyelinating diseases, such as multiple sclerosis (MS) (32).

ADEM symptoms may initially present after a prodromal period of several days or weeks (mean 12.5 days), which may not occur in MS (21, 33). A history of a precipitating event (previous infection or vaccinations) was not a prerequisite for the diagnosis of ADEM, which occurred in half of adult patients. The relationship between precipitating events and the occurrence of ADEM remains controversial, and possible mechanisms may include either molecular mimicry or direct inflammatory damage to myelinated neurons (34).Seasonal variation in the ADEM frequency (with peaks in spring and winter) supports its infectious aetiology (9). Patients with prior infection commonly had respiratory or gastrointestinal viral infections, such as rubella, mumps, measles, varicella, smallpox, and Epstein-Barr virus and infrequently had post bacterial infections, such as Mycoplasma pneumonia (35, 36). The severity of ADEM may be closely associated with different types of prior infection (37). A recent study included 30 ADEM cases after COVID-19 with a predominance of adult cases, possibly because adults are more likely to be infected by COVID-19 (38). For these adult cases, the average age was 50 years, which is older than those in previous reports of ADEM adults (39). More severe cases occur in older adults who often have multiple complications (40). Notably, ADEM can also occur in patients with asymptomatic COVID-19 infection (41). Postvaccination ADEM has occurred with various vaccines, including hepatitis B, hepatitis A, influenza, yellow fever, rubella, and poliomyelitis tetanus vaccines (22–24). In addition, a few recent studies reported that patients developed ADEM after being vaccinated for COVID-19 (42, 43). As postvaccination ADEM is rare, no definitive conclusions can be drawn about the association between a specific vaccine and the real risk of ADEM (44).

The clinical presentation is heterogeneous depending on the area involved in the demyelinating process. Four-fifths of the patients had a polyfocal clinical presentation associated with multifocal neurological deficits. Patients could show prodromal symptoms such as fever, headache, nausea, and vomiting. Fever was less common in adults than in paediatric patients, implying that age-related changes in the immune response, especially inflammatory cytokine reactions, might partially explain this difference (8). As fever is an unusual symptom in other demyelinating diseases, such as MS, this symptom may support the diagnosis of ADEM (21). The acute phase occurs with encephalopathy, which may be subtle, especially in the early course, and is often noted as “irritability” or “sleepiness” rather than confusion or obtundation (45). Studies in paediatric cohorts have shown encephalopathy, with rates ranging from 58.3% to 74% (8, 9, 18). In adults, the occurrence of encephalopathy is clinically important, as its absence may help identify other diseases, such as MS, which is less likely to present with encephalopathy (21). However, the frequency of encephalopathy in adults is relatively low (43.7%), suggesting that the absence of encephalopathy should not discourage neurologists from diagnosing this disease in adults. Encephalopathy is essential for the diagnosis of ADEM according to the IPMSSG criteria. These criteria may be overly restrictive for adults. We found that motor deficits, including hemiplegia, paraplegia and quadriplegia, were the most common clinical features, which occurred in more than three-fifths of patients, implying that pyramidal tract lesions are involved in most cases. Another common clinical feature is ataxia, which is rare in MS (46). Atypical symptoms, including meningeal signs, seizures and neuropsychiatric symptoms, may resemble CNS infectious diseases, highlighting a certain variability in the clinical presentation of ADEM. Thus, further auxiliary examinations including MRI imaging and CSF examinations are needed (5).

A previous study suggested that the clinical diagnosis of ADEM is mainly based on neuroimaging findings in clinical practice (47). MRI is a highly sensitive technique for detecting white matter abnormalities and is the preferred examination method for the diagnosis of ADEM (48). FLAIR and T2-weighted MR imaging is suitable for lesions detection. Lesions in most of ADEM patients show vasogenic cerebral edema on diffusion weighted imaging (DWI), which is beneficial to establish a differential diagnosis with other diseases (cytotoxic edema), such as acute cerebral infarction (49). ADEM is characterized by multifocal inflammatory demyelinating lesions in white matter (87.1%). Lesions are typically large and localized asymmetrically and have poorly defined margins. The deep grey matter is frequently involved (32.4%), often symmetrically, while cortical areas (23.8%) are involved to a lesser extent (29). The frequency of gadolinium-enhancing lesions is highly variable among studies, ranging from 30.0% to 95.2%, which may depend on the time of MRI evaluation. Notably, nearly one-tenth of ADEM adults present with a normal MRI or a delay of a few days to a few weeks between symptom onset and the appearance of MRI abnormalities (29). Thus, a normal MRI in the first days after symptom onset does not rule out a diagnosis of ADEM, and a follow-up MRI is needed for these patients. Compared with children, adults present with more frequent involvement of the periventricular areas and less often with involvement of the basal ganglia (8, 9). In addition, neuroimaging is useful to distinguish CNS demyelination diseases from other causes, such as MS and neuromyelitis optica spectrum disorder (NMOSD). Lesions in the thalamus and basal ganglia are more typical of ADEM than MS (50). Callosal lesions have been reported to occur in more than half of patients with MS (51, 52) but only in nearly one-fifth of adults with ADEM. Furthermore, a recent study compared callosal lesions on MRI among adults with CNS demyelinating diseases and found that radial lesions, defined as strip-shaped lesions spreading vertically from the lower surface to the upper surface of the corpus callosum, may be characteristic of MS and NMOSD but are rarely observed in ADEM (53). Moreover, a paediatric cohort study found that T1 hypointense lesions and more than two periventricular lesions may be MRI characteristics for distinguishing MS from ADEM, which requires further confirmation in an adult population (54). The spinal cord is also commonly involved in ADEM; however, an isolated spinal cord lesion without supratentorial involvement is unusual, which is different from NMOSD (55, 56).

CSF examinations in suspected ADEM should be performed rapidly after hospital admission to differentiate ADEM from other disorders, such as infection, MS and NMOSD. ADEM often presents as nonspecific inflammatory changes in most ADEM patients, including pleocytosis (51.8%) and elevated protein levels (39.1%) (29). Compared with the high incidence of CSF oligoclonal bands (OCBs) (>80%) in MS, positive CSF OCBs occurred in only one-fifth of ADEM adults (57). Among ADEM patients with positive OCBs, identical serum and CSF OCBs (‘mirror pattern’) were the most common pattern, suggesting predominant systemic immune activation, which is likely a consequence of infection or vaccination. However, most patients had CSF-restricted OCBs in MS. These results indicate that the detection of OCBs in both CSF and serum could help discriminate ADEM from MS (58). Autoantibodies targeting myelin oligodendrocyte glycoprotein (anti-MOG-IgG) are a marker of several central nervous system inflammatory demyelinating disorders, including ADEM, bilateral optic neuritis, transverse myelitis, and brainstem encephalitis. MOG-IgG positive children presented as ADEM in approximately 50% of cases, whereas adults in less than 10% (59). In children, high MOG-IgG at onset with declining antibody levels may be associated with a monophasic disease course and more likely to have a favorable long-term prognosis (60). While, persistent MOG-IgG may imply a recurrent disease course (61). Due to the limited ADEM cases in adults, further larger-scale longitudinal studies are needed to evaluate the value of MOG antibody in ADEM adults.

EEG is useful for the evaluation of brain function, especially if neuroimaging and CSF findings are negative (19). Diffuse background slowing is the most common finding, which may provide an alternative explanation for the encephalopathy observed in these patients (3, 26). In addition, the presence of focal slowing or epileptiform discharges may support seizures in ADEM patients.

Functional outcomes of ADEM in paediatric patients are generally favourable. Adults had a more unfavourable functional outcome and slower recovery: nearly one-tenth of adult patients died, and residual deficits occurred in nearly half of the patients during the follow-up. In addition, two-fifths of adult patients required admission to the intensive care unit (ICU). Moreover, the duration of hospitalization was longer for adults than for children (2, 9). The discrepancy in functional outcomes may be related to the reduced plasticity of the ageing brain and differences in immune responses, which has been hypothesized previously in a mouse model (62). Our analysis of the follow-up time-specifc mortality indicated that half of deaths occurred after the first 3 months. This finding stresses the importance of follow-up.

Previous studies suggested that impaired consciousness and seizures were associated with poor outcomes, while patients with fever, nonreactive CSF and a low lesion load on MRI were more likely to have a good outcome (8, 22, 26). However, due to the different risk factors and endpoints among these studies, it is difficult to merge and analyze the common risk factors among these studies. Moreover, recurrence may occur in adults, affecting 0% to 22.6% of patients according to different studies, and follow-up visits may be necessary (8, 18, 23, 25) As the prognosis of ADEM in adults is worse, more rapid and aggressive treatment may contribute to a better outcome in these patients (9). No randomized trials have identified the best treatment for ADEM, and small observational studies and expert opinions are the main basis for therapy. The most effective therapy for ADEM remains unclear. Based on the hypothesis of immune-mediated damage, the standard treatment is unspecific immunosuppressive therapy. High doses of corticosteroids are the first-line treatment and are used in most patients. Intravenous immunoglobulins (IVIGs) and plasma exchange (PLEX) are considered second-line treatment in patients with resistance or contraindications to steroids (3, 63, 64). Notably, PLEX could also be effective as rescue therapy in paediatric ADEM (65). Moreover, patients with contrast-enhancing lesions or mass effect may have the greatest benefit from PLEX (66). In addition, for a few patients with intracranial mass effects, decompressive craniectomy may be a lifesaving intervention for such cases (67, 68).

## Limitation

This study has several limitations. First, most of the included studies were retrospective with small sample sizes and some auxiliary examinations could not be available for all patients. Second, there were possible causes of heterogeneity among the included studies based on the inclusion of different races, studies conducted in different countries. The lack of continuity in the diagnostic criteria used throughout the past decades could also cause heterogeneity, an uniform diagnostic criteria is urgent, thus we proposed a summary of the key observations in ADEM adults. Fourth, only a few included studies evaluated prognostic factors, which should be investigated in further studies. Fifth, the examination of MOG antibody is absent in the original articles, which should be conducted in further studies. Finally, no randomized trials for the treatment of ADEM were found, and which treatment may be the best is unclear. Due to the limitations mentioned above, these data should be interpreted with caution, and further large and well-designed studies are needed to confirm the conclusions of this study.

## Conclusion

The outcomes of ADEM in adults are worse than those in children. Considering the differences in the clinical characteristics of ADEM between adults and children, to distinguish ADEM from other CNS demyelination diseases, diagnostic criteria specialized for adults are needed. In addition, controlled trials are needed to evaluate the effectiveness and safety of different immunotherapies for this disease.

## Data Availability Statement

The raw data supporting the conclusions of this article will be made available by the authors, without undue reservation.

## Author Contributions

JW, XD and KL had the idea for the study. KL, ML, LW and QW collected the data. All authors drafted and revised the paper, and approved the final version.

## Conflict of Interest

The authors declare that the research was conducted in the absence of any commercial or financial relationships that could be construed as a potential conflict of interest.

## Publisher’s Note

All claims expressed in this article are solely those of the authors and do not necessarily represent those of their affiliated organizations, or those of the publisher, the editors and the reviewers. Any product that may be evaluated in this article, or claim that may be made by its manufacturer, is not guaranteed or endorsed by the publisher.
